# Potential Antidiabetic, Antioxidative and Antiproliferative Properties of Functional Wheat Flour Muffins Enriched with White Clover Flowers (*Trifolium repens* L.)

**DOI:** 10.3390/ijms25189909

**Published:** 2024-09-13

**Authors:** Barbara Borczak, Agnieszka Szewczyk, Dominik Domagała, Joanna Kapusta-Duch, Teresa Leszczyńska, Marta Kotuła, Daniela Grulova

**Affiliations:** 1 Department of Human Nutrition and Dietetics, Faculty of Food Technology, University of Agriculture in Kraków, Al. Mickiewicza 21, 30-149 Krakow, Poland; 2 Department of Medicinal Plant and Mushroom Biotechnology, Faculty of Pharmacy, Jagiellonian University Medical College, 9 Medyczna St, 30-688 Krakow, Poland; 3 Department of Ecology, Faculty of Humanities and Natural Sciences, University of Prešov, 17th November St. 1, 080 01 Presov, Slovakia

**Keywords:** muffins, *Trifolium repens* L., organoleptic assessment, antioxidant properties, polyphenols, glycemic index in vitro, cytotoxic and antiproliferative activity

## Abstract

The aim of the study was to evaluate the functional properties of muffins fortified with white clover flowers (*Trifolium repens* L.), which were added to the dough in the following amounts: (*i*) 0% (control); (*ii*) 2.5%; (*iii*) 5.5%; (*iv*) 7.5%; and (*v*) 10%. The organoleptic properties were assessed by a panel of consumers. Additionally, the following parameters were also tested: basic chemical composition, total polyphenols, the antioxidant activity together with antiproliferative effects on the A375 melanoma cell line, starch nutritional fractions and the in vitro glycemic index. As a result, replacing wheat flour with white clover flour significantly affected the color, aroma and taste of the muffins. The content of proteins, fats, total ash, dietary fiber, resistant starch (RS), slowly digestible starch (SDS),total polyphenols and antioxidant activity increased statistically significantly with the elevated amount of white clover flour added to the dough. At the same time, the content of free glucose (FG), rapidly available glucose (RAG) and rapidly digestible starch (RDS), the value of the in vitro glycemic index and the viability of melanoma cancer cells decreased significantly. The muffins enriched with white clover flowers might constitute an interesting proposition and extension of the existing assortment of confectionery products.

## 1. Introduction

Diet has a major impact on human health due to the many nutrients, non-nutrients and chemical contaminants it contains that influence the proper body functioning. Moreover, the impaired balance of the body is nowadays mentioned as the main cause of body aging and the emergence of chronic non-communicable diseases, including cardiovascular problems, osteoporosis, obesity and cancer (some types), as well as impaired memory and other physical conditions. Recently, the World Health Organization (WHO) has stated that chronic non-communicable diseases (NCCDs), such as obesity and type 2 diabetes, will account for 74% of all deaths globally (World Health Organization, 2022) [1], while cancer is the leading cause of death worldwide and its prevalence is constantly growing. Based on cancer data in 2022, 1,918,030 new cancer cases and 609,360 cancer-related deaths are expected [2]. Therefore, there is a high need for functional foods, and the offer of healthier food options on the market is constantly growing [3]. Functional foods affect specific functions of the body, and may impart additional health benefits (other than basic nutrition) or cure certain diseases after the addition of a beneficial ingredient. Functional foods are defined as foods that have a visual appearance of the traditional kind and are a part of the daily diet. These products bring physiological benefits and/or can lower the risk of non-communicable diseases [4]. However, not all food products are equally suitable for supplementation or fortification to be successful as the supplemented foods should be consumed widely and in an adequate amount in order to play a beneficial role in the body. For this reason, baked goods such as bread or biscuits, but also pasta, are very frequently selected as the food matrix. In Europe, wheat and bakery products made from flour have traditionally been the basics of the diet [5,6]. A muffin is a sweet, high-calorie, popular wheat product that is baked and usually eaten during the day. In recent years, it has reached the status of a “snack” and is now eaten all over the world. Beyond the health benefits of functional foods, there are also other factors which contribute to the successful introduction of supplemented food in the food market. The economic aspect is equally significant. The production price should be reasonable and have little impact on the final price of the new product. Low cost of cultivation, a variety of different bioactive potential compounds and prevalent occurrence are the advantages of *Trifolium* species. Consumer acceptability is also a crucial parameter contributing to the feasibility of the products for launching and is strongly influenced by product tastiness, flavor and appearance [3,7]. The new product should meet the needs and expectations of consumers. Therefore, its acceptability must be evaluated.

Additionally, the taxa of *Trifolium (Leguminosae* or *Fabaceae*) are some of the most important genera of the *Leguminosae* family, both in terms of their agricultural value and the number of species (about 300), differing in their content of individual components with biological activity [8]. The genus *Trifolium* is found in the temperate and subtropical regions of both hemispheric continents and includes annual and perennial species. Many *Trifolium* species were originally native to central and southern Europe, North Africa, Asia Minor and China. Contemporarily, they are spreading to many other parts of the world where they have been accidentally introduced. However, many *Trifolium* species are cultivated extensively as fodder crops. Among most widely cultivated perennial species, *T. pratense* (red clover) and *T. repens* (white clover) are mainly mentioned, with the latter often considered as a highly important source of feed and food [8,9]. *T. repens*, also known as white clover, white trefoil, Dutch clover, creeping *Trifolium*, honeysuckle clover or ladino clover [9], has a characteristic structure. The stem of it is creeping, up to 45 cm long, leafless, smooth and full. The leaves are trifoliate, long-petioled and obovate, with serrated edges. There is a light horseshoe-shaped spot on the upper side of the leaf. The flowers grow in the form of a spherical head growing on a long peduncle, containing 40–80 self-pollinating papilionaceous flowers. The seeds are small, heart-shaped and light or dark yellow in color, with a shine. In traditional medicine, *Trifolium* species were used for a long time by many cultures and, more currently, by Americans in the treatment of eczema, psoriasis and external skin, lung, nervous and reproductive problems. The application of white clover as a deworming remedy by the Naga tribes of India was also reported [8]. It has also been evidenced that the isoflavones of red and white clovers have a similar structure to 17-β-estradiol (a female sex hormone) and hence may have an estrogenic activity [10]. The three major kinds of phytoestrogens are isoflavones, lignans and coumestans [8]. Christiansen et al. [11] reported eight different isoflavones in *T. repens* including biochanin A, genistein, formononetin, sissotrin, puerarin, daidzin and daidzein. They are proved to have a positive influence on menopausal disorders such as breast cancer, bone health, cardiac risk factors, osteoporosis and endothelium-dependent vasodilation in postmenopausal women. In addition to their estrogenic activity, phytoestrogens may exhibit nonhormonal functions such as antioxidant effects with possible anticancer properties. In some countries, especially the US and EU countries, dietary supplements containing this plant are available on the market. In the mountainous regions of Pakistan, extracts of or drinks made from the plant *T. repens* are used to treat joint injuries, coughs, colds, fever, sore throat infections or abdominal pain [9]. White clover may also contain cyanogenic glucosides and saponins. These compounds were evidenced to have antifungal and antidiabetic (cyanogenic glucosides), hepatoprotective, anti-inflammatory and antioxidant (saponnins–soyasapogenol B) properties and are toxic to nematodes.

Considering the above, the aim of the study was to evaluate the potential antidiabetic, antioxidative and antiproliferative properties of muffins fortified with white clover flowers (*Trifolium repens* L.).

## 2. Results

### 2.1. Organoleptic Evaluation of Muffins with White Clover Flowers (Trifolium repens *L.*)

The control muffins and those with an addition of 2.5% and 5% white clover flowers were assessed as the best, obtaining 7.95 points, 7.84 and 7.78, respectively, on a nine-point hedonic scale (*p* < 0.05) (Table 1). Those with a 7.5% addition of *Trifolium repens* L. also received a good score (7.51 points) and did not differ in a statistically significant way from the muffins with 5% enrichment (*p* > 0.05). The overall rating of the muffins with the highest addition of the plant (10%) was lower (6.82 points) in a statistically significant way (*p* < 0.05) and received moderate acceptance. 

### 2.2. Basic Chemical Composition of the Tested Muffins

The results of the chemical composition studies of both *Trifolium repens* L. in a powdered form and muffins supplemented with the plant are presented in Table 2. The addition of growing amounts of the flower powder (above 2.5%) to the muffins resulted in a statistically significant increase in all nutritional components, as shown in Table 2 (*p* < 0.05).

### 2.3. Starch Nutritional Factions and the Value of the In Vitro Glycemic Index

**Figure 1:**
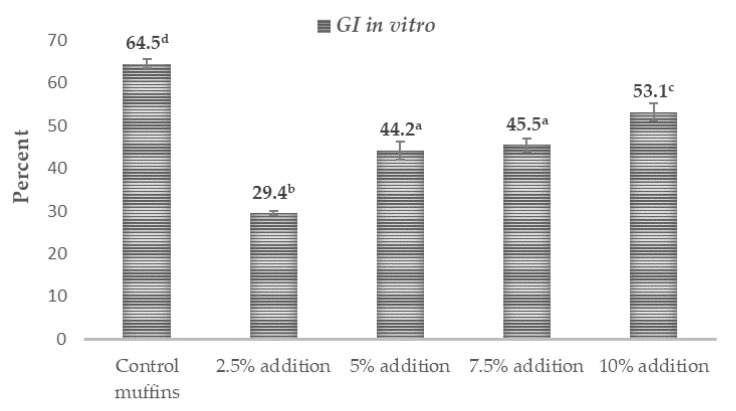
Potential antidiabetic effects of muffins containing white clover flowers (*Trifolium repens* L.). The results are presented as mean ± sd. The values with varied letters are significantly different at *p* < 0.05. Starch digestibility index (SDI) is referred to as ‘GI in vitro’.

The results relating to the possible in vitro glycemic properties are presented in Table 3 along with Figure 1 and show a very promising influence of *Trifolium repens* L. on the probable antidiabetic characteristics of the supplemented muffins. The amount of total starch (TS) in the muffins was in the range of 42.04–43.85 (g·100 g^−1^ dm). The addition of white clover flowers at the level of 5–10% contributed to a higher value of this compound compared with the control muffins and those with 2.5% supplementation (*p* < 0.05). The amount of rapidly digestible starch (RDS) in the tested muffins was between 9.88 and 22.03 (g·100 g^−1^ dm). All white-clover-flower-enriched muffins were characterized by a significantly lower content of this fraction compared with the control samples (*p* < 0.05). The content of slowly digestible starch (SDS) was between 3.7 and 6.73 (g·100 g^−1^ dm), while the observed resistant starch (RS) was in the range of 16.54–28.57 (g·100 g^−1^ dm) (Table 3). The values of these indicators were statistically significantly higher in all the white clover flower muffins (*p* < 0.05). The calculated values of the starch digestibility index (SDI) referred as to glycemic index in vitro (GI in vitro) in the muffins were between 29.44% and 64.45% dm (Figure 1), while rapidly available glucose (RAG) (the sum of free glucose and the glucose released after 20 min of in vitro digestion) and free glucose (FG) were at the level of 19.86–34.67 (g·100 g^−1^ dm) and 4.43–5.09 (g·100 g^−1^ dm), respectively (Table 3). Again, all the muffins reinforced with the flowers of *Trifolium repens* L. were characterized by statistically significantly lower amounts of those parameters compared to the control muffins (*p* < 0.05).

### 2.4. Antioxidant Properties of the Muffins

**Figure 2:**
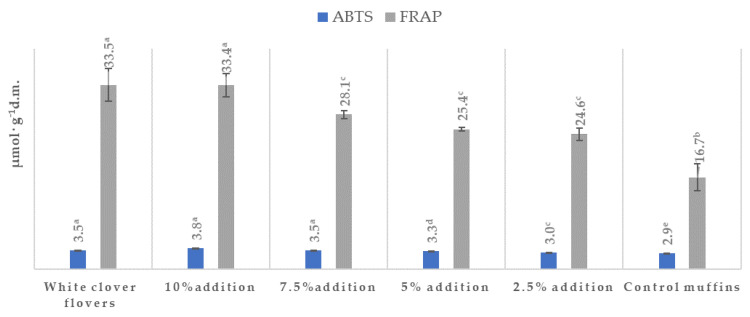
Antioxidant effects of muffins containing white clover flowers (*Trifolium repens* L.). The results are presented as mean ± sd. The values with different letters are significantly different at *p* < 0.05. Results are according to ABTS (2,2-azinobis) (3-ethyl-beznothaizoline-6-sulphonic acid) and FRAP (Ferric-Reducing Ability of Plasma) methods.

**Figure 3:**
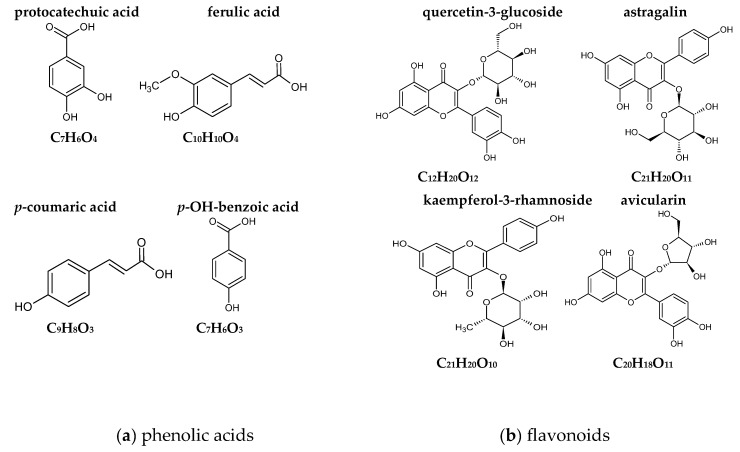
Chemical structures of the assayed phenolic compounds detected in muffins supplemented with white clover flowers (*Trifolium repens* L.).

The results of the antioxidant activity determinations and the amounts of the particular polyphenols are presented in Table 4 and Figure 2, while their chemical structures are shown in Figure 3. The content of total polyphenols was in the range of 21.26–431.37 (mg·100 g^−1^ dm) for the muffins and 2409.36 mg for the lyophilized white clover flowers. The content of total polyphenols was statistically significantly highest in the muffins with the addition of 10% (431.37 mg) and 7.5% (165.31 mg) *Trifolium repens* L. (*p* < 0.05), while, in the case of the muffins with the addition of 2.5%–5%, it was in the range of 232.65–292.94 mg (Table 4). The statistically significantly lowest content of these compounds was found in the control muffins (21.26 mg) (*p* < 0.05). The fortified muffins were characterized by a statistically significantly higher (up to 20 times greater) number of total polyphenols than the control muffins (*p* < 0.05). Among the detected molecules, phenolic acids and flavonoids were predominant, but the latter were assayed only in the lyophilized flowers of *Trifolium repens* L. and in the supplemented muffins. In the control muffins, no flavonoids were observed (Table 4). Among the acids, two were hydroxycinnamic acids, namely, *p*-coumaric and ferulic acids, while the other two were classified as hydroxybenzoic acids (protocatechuic acid and *p*-OH-benzoic acid). All phenolic acids were found in the supplemented muffins, with an increasing number correlating with the growing percentage share of the flowers in the muffins; however, *p*-coumaric was not detected in the control muffins. The antioxidant activity of the muffins was between 2.86–3.82 μmol trolox· g^−1^ dm and 16.72–33.42 μmol Fe (II)· g^−1^ dm, as measured by the ABTS and FRAP methods, respectively (Figure 2). Irrespective of the measuring method, the antioxidant activity of the white-clover-flower-enriched muffins was greater than that of the control muffins (*p* < 0.05) and became enhanced upon increasing addition of *Trifolium repens* L. flowers. Additionally, the antioxidant activity of the lyophilized white clover flowers was at the level of 3.48 μmol trolox· g^−1^ dm and 33.57 μmol Fe (II)·g^−1^ dm, as measured by ABTS and FRAP methods, respectively. Interestingly, there were no statistically significant differences between the antioxidant potential of the raw material and the muffins with 7.5% and 10% addition (*p* > 0.05) (Figure 2).

### 2.5. Cytotoxic and Antiproliferative Properties of the Tested Muffins

**Figure 4:**
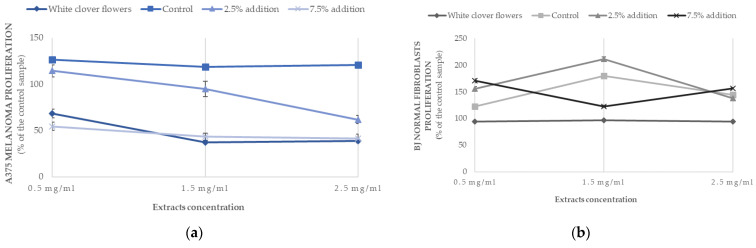
Antiproliferative effects of muffins containing white clover flowers (*Trifolium repens* L.) on (**a**) melanoma A375 and (**b**) human normal fibroblasts BJ cell lines depending on the extracts’ concentration (0.5 mg/mL; 1.5 mg/mL; 2.5 mg/mL). The results are presented as mean ± sd.

The cytotoxicity and proliferation rate were tested in the selected muffins, including the control muffins and those with an addition of 2.5% or 7.5% of white clover flowers, in order to see the difference between the lowest and highest doses of the plant. The *Trifolium* extract from the flowers was also included in the experiments. When analyzing the effect of different extracts on the cytotoxic properties, the recorded values were equally low (approximately at the level of 12–15%) independent from the tested cells (skin cancer A375 or BJ normal fibroblasts), indicating no cytotoxic effect of the tested extracts (Table 5). The anticancer properties of the tested extracts were better seen when proliferation rate was examined (Table 5, Figure 4). The viability of melanoma cells was inhibited by almost 50% when extracts from *Trifolium repens* L. and the muffins with 7.5% addition of the flowers were used. When the extracts of the muffins with 2.5% addition and the control muffins were tested, the proliferation rate of melanoma cells was much higher, ranging from 90 to 122% (Figure 4), indicating that the greater addition of the flowers (7.5%) was more effective in reducing the cancer cells’ growth. When the same extracts were tested on normal BJ fibroblasts cells, the proliferation was very high (95%–148%) and might suggest that the extracts were safe for normal cells.

## 3. Discussion

In the literature, there were no data on the organoleptic assessment of muffins with white clover flowers. The overall sensory attributes of the muffins enriched with other plants’ by-products recorded by other authors seem dependent on the amount of incorporation, which alters their consumer acceptance: wheat grass powder (7.5%) [12]; white and red grapes pomace [3], garden cress [13] (upon 20% addition); cauliflower (20–30% addition) [14]; and fresh ginseng (with increasing loading up to 10–40%) [7]. In most supplemented muffins, a decrease in all attributes including appearance, taste, aroma, texture, softness, palatability, color and overall acceptability was observed. In this study, the flavor and elasticity were assessed as worse in the case of muffins with 10% white clover addition in comparison to the other ones (*p* < 0.05), while the tastiness of all muffins differed among all tested kinds, being the most tasty (“strongly like”) in the case of the control samples and the sample with 2.5% addition, “moderately like” when 5% and 7.5% enriched muffins were tested and “slightly like” for the 10% addition (Table 1). An interesting point is that the shape of all flower-enriched muffins was similarly approved by the testers, while the color of the 10% addition muffins was not accepted as much as that of the other tested muffins (described as “like a bit”) (*p* < 0.05). 

To our best knowledge, there are no data in the literature concerning the effect of white clover flower (*Trifolium repens* L.) additions to wheat flour muffins on the basic nutritional composition. However, the obtained results are in concordance with the data presented in the literature for wheat-flour muffins in the case of dry matter—57.12–85.60 g∙100 g^−1^, carbohydrates—35–98.75 g∙100 g^−1^, protein—6.37–16.34 g·100 g^−1^ dm, fat—10.02–26.70 g·100 g^−1^, ash—1.19–4.0 g·100 g^−1^ and dietary fiber contents—1.2–5.67 g·100 g^−1^ [3,5,6,7,13,15,16,17,18]. The enrichment with increasing amounts of the flower powder (more than 2.5%) contributed to the statistically significant increase in some nutritional components, including proteins, dietary fiber, fats and ash (Table 2) (*p* < 0.05). In the literature, there is scarce information on the proximate composition of white clover flowers. In the found studies, it was reported that the flowers of *Trifolium repens* L. are rich in proteins (14–28%) and crude fiber at the level of 20.6–31.4% [19,20], and the recorded differences were most probably due to various habitat conditions. The percentage share of starch in the muffin batter, containing flour, sugar, fat, eggs and baking powder, depends strongly on the recipe used for the cupcake preparation [21]. Upon baking, complex reactions take place and involve water evaporation, protein denaturation, starch destruction, browning and Maillard reactions, dough and thermal expansion of gas. In the presence of water and high temperature, the starch granules swell and the hydrogen bonds between the amylose chains in the structure of the starch break down. Part of the amylose is released from the granule, forming spaces into which the water enters. These changes are called gelatinization, which leads to the loss of the starch crystalline structure [22]. Consequently, the structure of the starch is less concise and more accessible to digestive enzymes. Rapidly digestible starch (RDS) is defined as a ‘type of starch which is quickly (within 20 min) converted to glucose molecules by enzymatic digestion’. If RDS is present in high proportions in food, it will rapidly release glucose into the blood and thereby elevates blood glucose and insulin response, which is detrimental to health. RDS is significantly correlated with the glycemic index, based on the in vivo postprandial glycemic index [23], and might be a useful predictor of the probable glycemic response when tested by in vitro methods. The amount of RDS in the wheat flour muffins mentioned in the literature was between 33.8 and 44.4 (g·100 g^−1^ dm). The RDS contents obtained in this study were below the range proposed by the other authors in the control sample (22.03 g·100 g^−1^ dm) [6,24] and were even more reduced, by 29–55%, when the flowers of *Trifolium repens* were included (Table 3). In the available literature, there is no information on the content of RDS in wheat flour muffins enriched with white clover flowers. The RDS amount in foodstuffs represents the realistic level of easily digestible starch, which is primarily responsible for the short-term postprandial glycemic response, whereas SDS refers to the slowly digested starch fraction, which provides a low and prolonged glycemic response because it is completely converted into glucose in the small intestine but over a longer time (after 120 min) [22,24]. Interestingly, the fractions of slowly digestible starch (SDS) and resistant starch (RS) were also affected by the addition of *Trifolium repens* flowers. SDS was significantly higher in the muffins with 5–10% addition (*p* < 0.05), while RS was the greatest when 2.5% flowers was incorporated into the muffin butter (*p* < 0.05). Thus, the reducing effect on starch digestibility was observed in the white clover flower muffins. The SDS presented by other authors was in the range of 3.15 to 4.5 (g·100 g^−1^ dm) [6,24], and biscuits are classified as food products with low RS (1.5%–2.5) [25]. In the case of SDS, the values were within the range provided by the literature for the control and 2.5% supplemented muffins, and greater amounts of *Trifolium repens* L. (5%–10%) resulted in data values higher than those in the literature. The recorded results for resistant starch were much higher than the literature data for this kind of food product (Table 3). The possible explanation might be the development of the insoluble complex of linear poly-*α*-1,4-glucan formed by the different lipids present in the muffinswith starch molecules resistant to degradation by *α*-amylase, which leads to the formation of newly classified resistant starch called RS5. These polysaccharides facilitate the formation of short-chain fatty acids (SCFAs), particularly butyrate, which is the most important SCFA [23]. The amylose–lipid complex induces the formation of ordered structures to modulate starch digestibility, thereby limiting the swelling of starch and accessibility of digestive enzymes. Lipid–amylopectin interactions also occur, but the binding strength of the amylopectin–lipid complex is weaker than that of the amylose–lipid complex [26]. The digestibility of starch and hence the contents of the starch fractions (RDS, SDS and RS) in food products might be dependent on factors that can generally be classified as intrinsic (crystal type, granular structure of the starch, amylose-to-amylopectin ratio, the contents of lipids, proteins, polyphenols or dietary fiber), external (processing methods and storage conditions) or exogenous, such as food ingredients and additives (amino acids, lipids, salts, sugars, gums, etc.) [26]. In this study, an addition of lyophilized flowers of white clover in the amount of 5–10% contributed to a reduction in RDS of 29–56% and in GI in vitro (SDI) of 18–55% and led to an increase in SDS of 28–80% and in RS of 12–16% (Table 3, Figure 1). This is a very favorable phenomenon from a nutritional point of view. It has been shown that the occurrence of RS in food products may have health-promoting effects on living organisms, i.e., an increase in the amount of short-chain fatty acids (SCFAs), a reduction in the pH of the colon, an increase in the growth of probiotic bacteria and a reduction in postprandial blood glucose and blood cholesterol levels [23]. A possible reason for the increased content of SDS and RS in all white-clover-supplemented muffins, with a simultaneous decrease in the content of RDS and GI in vitro (SDI), might be the presence of polyphenolic compounds and dietary fiber. The contents of these components were statistically significantly increased in the *Trifolium repens* L. muffins (*p* < 0.05) (Table 2 and Table 3).

### 3.1. Possible Antidiabetic Mechanisms and Pathways

It has been evidenced that polyphenols constitute a useful tool for the modification of starch digestibility and may act in several possible ways. First, the activities of amylolytic enzymes (α-amylase and α-glucosidase) have been found to be inhibited by polyphenols, thus retarding the rate of digestion. The kind and number of polyphenols play a crucial role in this suppressive effect. Monomeric phenols inhibit enzymes primarily by blocking catalytic sites, whereas polymeric phenols can bind to enzymes to increase digestive resistance [27,28]. Second, in proper concentrations, polyphenols can interact with other food compounds to cover the starch granules and reduce their rate of digestion [29]. There is also another possibility, in which linear amylose itself interacts with hydroxyl groups of polyphenols to create non-inclusion complexes through interactions involving hydrogen bonds, hydrophobicity, electrostatics and ionic interactions [30]. Moreover, V-type inclusion might be formed, in which polyphenols are partly encapsulated in the hydrophobic pockets of starch [31]. These inclusion complexes facilitate the formation of resistant starch (RS5), thereby limiting starch digestibility. They can also slow down the process of digestion by reducing the starch swelling and creating a more compact starch network with reduced enzyme accessibility [32]. The glycemic indices expressed as starch digestibility indices were significantly lower in all the white clover muffins compared with the control sample, by 18%–54% (Figure 1) (*p* < 0.05). Generally, biscuits are food products with a moderate-to-high glycemic index due to their sugar and refined wheat flour content [6,33,34]. The intake of high-glycemic-index/load foods and diets is correlated with an enhanced risk of developing chronic, non-communicable diseases such as type 2 diabetes, obesity, coronary heart diseases or certain types of cancer [17,21,22]. Currently, muffins are experiencing a surge in demand in both Western and emerging markets [35]. Therefore, the provision of healthier muffin alternatives with a lower glycemic index than conventional products could be a strategy to improve public health [1]. The glycemic indices expressed as the starch digestibility index of all the white clover flower muffins were very low (below 55%) (Figure 1). In the literature, there was no information about the glycemic index of muffins with *Trifolium repens* L. flowers. However, some authors reported the antidiabetic properties of white clover, supported by the studies, mainly by the presence of secondary metabolites such as cyanogenic glycosides [9] and polyphenols [36,37]. All of the muffins contained different polyphenolic compounds from both subcategories, phenolic acids (ferulic, protocatechuic, benzoic and *p*-coumaric) and flavonoids (quercetin 3-glucoside, quercetin-3-α-l-arabinofuranoside, kaempferol-3-O-glucoside, kaempferol 3-rhamnoside) (Table 4, Figure 2 and Figure 3). All of the reported flavonoids and *p*-coumaric acid were found in the muffins supplemented with *Trifolium repens* L. and were not detected in the control muffins. In the recent literature data, promising findings have been documented concerning the role of the selected polyphenolic compounds in the treatment of type 2 diabetes. 

Polyphenolic molecules may provide antidiabetic benefits targeting glucose homeostasis in different ways [27,28,29,30,38,39,40,41,42,43,44,45,46,47]. First, they might contribute to the protection of pancreatic ß-cells against oxidative damage and cell apoptosis, stimulate ß-cell regeneration, reduce oxidative stress and control insulin-dependent and -independent signaling pathways. Second, they can participate in the modification of glycogenesis and glycolysis or provide the inhibition of alpha-amylase and alpha-glucosidase activities. It has also been evidenced that these molecules are able to modify the transport of glucose through biological membranes by the modification of both intestinal (SGT2, SGLT2) and adiposal or skeletal (GLUT4) transporters. For instance, quercetin-3-glucoside improved glucose uptake in insulin-resistant muscle cells up to 1.2 fold in free-fatty-acid-induced insulin-resistant C2C12 myotubes over a concentration range of 2–54 µM (1–25 µg/mL) [38]. It has been found that this compound can improve glucose metabolism through upregulation of the SIRT1/AMPK/GLUT4 signaling pathway. In more detail, it showed stable interactions with SIRT1 (the nicotinamide-adenine-dinucleotide-dependent enzyme sirtuin 1) residue through the formation of hydrogen linkages upon molecular docking, stimulates SIRT1 upregulation and AMPK (5′-adenosine monophosphate-activated protein kinase) phosphorylation and upregulates GLUT4 translocation [38]. The activation of sirtuin, a nicotinamide adenine dinucleotide (NAD+)-dependent histone deacetylase, is proposed as an innovative therapeutic means, and it plays an important role in inhibiting the progression of insulin resistance in insulin-sensitive tissues along with protecting the pancreatic ß-cells. The antidiabetic potential of quercetin-3-glucoside in streptozotocin-induced diabetic Wister rats has been reported by Jaychandran et al. [39] and Zhang et al. [40], acting as a dipeptidyl peptidase-4 inhibitor in the glucagon-like peptide (GLP)-1 signaling pathway. Additionally, an in vivo study on rat livers suggested that *p*-coumaric and ferulic acids can inhibit gluconeogenesis. Elevated gluconeogenesis and glycogenolysis lead to the declined levels of hepatic glycogen and increased blood glucose concentration which have been reported in individuals with type 2 diabetes. This is due to the continuous activation of the pathways’ key enzymes resulting in increased activities of glucose 6-phosphatase, fructose 1,6-biphosphatase and glycogen phosphorylase in the hepatic tissues. The decreased activities of these enzymes and simultaneous elevated hepatic glycogen level were reported in ferulic-acid-treated rats [41] and in *p*-coumaric streptozotocin-induced rats (100 mg/kg body weight) [42] and therefore suggest the activation of glycogenesis and glycolysis, which contribute to hypoglycemia and decreased availability of glucose. *p*-Coumaric was also evidenced to have a protective role against diabetic nephropathy through its anti-inflammatory and antioxidant properties [43], was proved to reduce TLR 4, IL-6 and TGF levels when applied at a dosage of 100 mg/kg body weight and increased phosphorylation of acetyl CoA carboxylase (ACC) as well as the expression of carnitine palmitoyltransferase-1 (CPT-mRNA) and peroxisome proliferator-activated receptor (PRAR), which may result in the elevated beta-oxidation of fatty acids and triacylglycerol synthesis [44].

According to the literature, the total polyphenol content (calculated in mg of gallic acid·100 g^−1^ d.m) of cereal-based products and wheat flour is in the range of 28.3–344 [48,49]. The results of this study are consistent with this range, except for the results for the control muffins, which were below this range, and for muffins supplemented with 10% flower, which were above the values proposed by the other authors. The data on total polyphenol content in *Trifolium* species are very poor and mostly concern other species, namely, *T. pratense* or different grasslands plants. The content of polyphenols was in the range of 3438–15,250 (mg of gallic acid/100 g), in which total flavonoid content was 1262–2184 mg catechin equivalent/100 g [50,51,52,53], while 4980 (mg of gallic acid/100 g) of total polyphenols and 820 (mg rutin equivalent/100 g) of flavonoids were found in the flowers of *T. repens* [54]. In the thematic literature, there is a lack of information on the number of total polyphenols in muffins with *Trifolium repens*. Phenyl propanoic acids (caffeic, *p*-coumaric, synapic, ferulic) and phenolic acids (vanillin, *p*-hydroxybenzoic, protocatechuic, salicylic, gallic and ellagic acids) are the major polyphenols in cereals. In cereal grains, the principal phenolic acid is trans-ferulic acid [55]. In wheat grains, insoluble fractions predominate (77%), followed by bound soluble acids (22%), including free and soluble fractions (0.5–1%) [56]. Baking at high temperatures (> 200 °C) can have a significant effect on polyphenolic compounds, leading to some structural changes [57]. For instance, flavonoids tend to be more stable during heating than anthocyanins, while baking enhances the number of free phenolic acids in bread, cakes and muffins but reduces the quantity of bound phenolic acids [58], indicating that baking may favor the delivery of phenolic acids from bound to free form.

This study reported the presence of astragalin (61.48 mg/100 g), also known as kaempferol-3-O-glucoside, kaempferol–3-rhamnoside (25.96 mg/100 g) and quercetin–rhamnoside (371.27 mg/100 g). Additionally, one more compound—avicularin (36.08 mg/100 g), also called quercetin-3-α-l-arabino furanoside—was not presented by the other authors (Table 4, Figure 2 and Figure 3). The differences in the flavanol contents of *T. repens* between the data from this study and those reported in the literature might be due to the various cultivars studied by Hofmann et al. [59], who showed different flavonol contents in miscellaneous parts of plants. For example, flavonol contents in leaves were 2–1700 mg/g, in seeds 2.8–2000 mg/g, in total above-ground materials 20–2210 mg/g, in roots 208 mg/g and in flowers 66–481 mg/g.

The presence of the above mentioned flavonoids was not found in the control muffins in our study; however, due to the enrichment with the lyophilized flowers, the muffins gained more bioactive compounds, adding a very beneficial effect. 

Moreover, the antioxidant activity of the white clover flower muffins was greater than that of the control muffins (*p* < 0.05) irrespective of the measuring method and became considerable higher upon increasing addition of *Trifolium repens* L. flowers to the muffin batter (Figure 2). The antioxidant activity of the lyophilized flowers was at the level of 3.48 μmol trolox·g^−1^ dm and 33.57 μmol Fe (II)·g^−1^ dm, as measured by ABTS and FRAP methods, respectively. Interestingly, there were no statistically significant differences between the antioxidant potential of the raw material and muffins with 7.5% and 10% addition (*p* > 0.05) (Figure 2). A limited body of evidence exists relating to the phytochemical and antioxidant properties of *Trifolium* species, which are outlined in Table 4 and Figure 2. The FRAP values were in the range of 89.54–136.05 μmol Fe (II)·g^−1^ dm in *Trifolium pratense*, while, in the case of other grassland plants such as *Lolium perenne* L., *Cichorium intybus* L. and *Plantago lanceolata* L., they were in the range of 67.36–896.68 μmol Fe (II)·g^−1^ dm [10]. According to the available data on *T. repens*, the flower extracts exhibited a promising ferric-reducing power with an FRAP value of 44.2 μM Fe(II)/g and 21.4 ± 2.5 IC50 (μg/mL) or 21.4 µg/mL [60,61,62] as measured by ABTS method. For comparison, the ABTS antioxidant power of ascorbic acid was 1.7 ± 0.3 IC50 (μg/mL), while the FRAP antioxidant potency of buthyl-hydroxy-toluene (BHT), an artificial antioxidant used as a common food additive in different foodstuffs, was at the level of 63.2 ± 4.3 μM Fe(II)/g [62]. The published data report variations within antioxidant activity. The dissimilarity may be caused by a number of reasons, including the variety of cultivars and growing locations, the origin of the plant, the part of the plant used (aerial or root) and the environmental growth features. These factors affect the occurrence of plant secondary metabolites (including polyphenols) and their biological activity [10]. 

### 3.2. Potential Antioxidant Mechanisms and Pathways

White clover flowers exhibit promising antioxidant potential, and, by the utilization of this plant, eight different polyphenolic molecules (four phenolic acids and four flavonoids) were reinforced in the muffins, which facilitated the free-radical-scavenging ability as compared to that of the control muffins (*p* < 0.05). Reactive oxygen species (ROS) are side products of aerobic metabolism. The excessive production and consumption of these reactive molecules may destroy redox balance in vivo and facilitate or inactivate numerous signaling pathways, as well as modulate transporters, ion channels and other signaling molecules such as protein kinases and ubiquitin/proteasome-system-related signaling molecules [63,64]. Due to their antioxidant activity, polyphenols can potentially protect DNA and lipids from oxidation through reactive oxygen species, and this has therapeutical meaning in oxidative-stress-related diseases such as Alzheimer’s, diabetes, skin cancer, hypertension and atherosclerosis.

Flavonoids, as much as a number of other plant polyphenols, have a chemical conformation ideal for the scavenging of free radicals. Their antioxidant characteristics involve the ability to interact with a wide range of reactive oxygen species, as well as metal chelation or inhibition of pro-oxidative enzymes [65,66]. Quercetin-3-glucoside was present in the white clover flowers (371.265 ± 17.85 µg·100 g^−1^ dm) and hence in the muffins in the biggest amount, while *p*-coumaric acid was detected in the muffins supplemented with flowers but not in the control muffins (Table 4, Figure 2). The observed differences may result predominantly from the various chemical structures which these molecules have (Figure 3). It has been found that the most important factors are the degree of methylation and the number of -OH groups [66,67,68,69,70]. 

### 3.3. Possible Anticancer Mechanisms and Pathways

To our best knowledge, this is the first study that investigates the anticancer activity of extracts obtained from wheat flour muffins with an addition of lyophilized flowers from *Trifolium repens* L. at three different concentrations, 0.5 mg/mL, 1.5 mg/mL and 2.5 mg/mL, on the A375 melanoma cancer cell line (Table 5, Figure 4).

Different natural substances and their compounds are of great interest nowadays not only in the prevention of cancer, but also as a supportive tool in traditional medicine [2].

There are limited literature data on the biological compounds in *T. repens* and their medicinal evaluation, including evaluation of their anticancer and antioxidant properties, especially the difference between the varied morphological parts of this plant (stalk, leaves, flowers) [9]. In this study, the viability of A375 human melanoma cancer cells was inhibited by almost 50% when the extracts from *Trifolium repens* L. and muffins with 7.5% addition of the flowers were used (Table 5) (Figure 4), indicating that the greater addition of the flowers (7.5%) was more effective in reducing the cancer cells’ growth. When the same extracts were tested on normal BJ fibroblasts cells, the proliferation was very high (95%–148%), which might suggest that the extracts were indifferent to normal cells, even promoting their growth. Similar research showed that extracts from *T. repens* had a cytotoxic effect on A549 cell lines [71], while Sarno et al. [72] tested the antitumor potential on a variety of liquid and solid cancer cell lines, including colon cancer HCT-116, breast cancer MCF7, lung cancer A549 and hepatocellular carcinoma HepG2 cells, though an effect was only observed in chronic myeloid leukemia (CML) cells. The possible mechanisms responsible for this phenomenon are the inhibition of BCR-ABL/STAT5 and activation of the p38 signaling pathway provided by the *T. repens* extracts. These potent cytotoxic effects were not observed in normal cells. There is scarce literature on the anticancer activities of *T. repens* extracts. However, the single polyphenolic compounds found in the white clover flowers were proved to demonstrate different anticancer properties [9]. RP-HPLC analyses identified the presence of protocatechuic acid, ferulic acid, *p*-OH benzoic acid, *p*-coumaric acid, kaempferol–3-glucoside (astragalin), kaempferol–3-rhamnoside, quercetin–rhamnoside and quercetin-3-α-l-arabino furanoside (avicularin) in different proportions (Table 4) (Figure 2 and Figure 3). Flavonoids and *p*-coumaric acid were present only in supplemented muffins and were not found in control samples in this study.

It is well known that the skin is the organ most exposed to environmental factors, and millions of people are affected by skin cancer every year [73,74,75,76,77]. This was the reason why melanoma was chosen to study the anticancer properties of the white-clover-enriched muffins. Melanoma is the most aggressive skin cancer, and, at the same time, has the highest frequency of mutations and the capacity to evade the immune function, and is known to be metastatic and lethal [74,75]. Although melanoma accounts for only 3% of all skin cancers, it is associated with the highest mortality rate (around 65%) [77]. An appropriate daily diet is a great chemopreventive tool for maintaining health and well-being. Therefore, healthier alternatives to the traditional muffins currently proposed on the food market which reduce the potential growth of melanoma cells could be an interesting proposition in daily dietary prevention.

It has been documented that polyphenolic molecules may act in melanoma cancer prevention and treatment, involving different mechanisms and pathways such as cytotoxicity, inactivation of angiogenesis, cell cycle arrest, autophagy, tumor metastasis, protection against UVB radiation and inhibition of inflammation, which is important since around 25% of human cancers are associated with chronic inflammation, as well as bacterial/viral infections. Other mechanisms include the activation of tumor suppressor genes and, furthermore, playing a significant role in different cell signaling pathways. The anticancer activity of flavonoids has been ascribed to their capability to chelate the transition metals (Fe^2+^, Fe^3+^, Cu^2+^) involved in free-radical-generating reactions, thereby modulating oxidative stress [47,48,66,68,73,74,78,79,80,81,82,83,84,85].

In a study by Amboth [78], ferulic acid significantly reduced the occurrence, weight and volume of UVB-promoted tumors in the skin of mice as compared to control animals. It was also seen that ferulic acid treatment could reverse the chronic oxidative deterioration of UVB-induced skin tumors in mice while modifying the expression of inducible nitric oxide synthase (iNOS), tumor necrosis factor-α (TNF-α), vascular endothelial growth factor (VEGF) and interleukin (IL-6). Ferulic acid application also modified the expression of mutated tumor protein 53 (p53), B-cell lymphoma-2 (Bcl-2), and Bcl-2-associated X (Bax) in UVB-induced mouse skin tumors. The results showed that ferulic acid had a promising inhibitory effect on UVB-induced carcinogenesis in albino mice. At the same time, *p*-coumaric acid was also evidenced to minimize the oxidative stress in keratinocytes exposed to ultraviolet radiation [68], together with kaempferol and its derivates, which exerted a cytostatic effect against A431 skin cancer [47]. Kaempferol was also proved to demonstrate significant antiproliferative effects on human malignant melanoma A375 cells, contributed to the reduction in colony formation in a dose-dependent manner, induced apoptosis, exhibited the capacity to trigger G2/M cell cycle arrest, suppressed the cell migratory potential of A375 cells and caused significant downregulation of the mammalian target of rapamycin (m-TOR), phosphorylated (p) m-TOR, phosphatidylinositol-3 kinase (PI3K) and phosphatidylinositol-3 kinase (PI3K)/Akt protein levels in A375 malignant melanoma cells [79]. Simultaneously, quercetin slowed down the invasion and mobility of murine melanoma B16-BL6 cells in a dose-dependent manner, obstructed the proliferation of B16-BL6 cells and dose-dependently reduced the cell rates in the S and G2–M phases of the cell cycle. Furthermore, quercetin significantly inhibited the expression of anti-apoptotic protein Bcl-2. [80]. An intraperitoneal dosage of quercetin and the antiestrogen tamoxifen, at the time of injection of B16-BL6 cells into syngeneic mice, resulted in a significant, dose-dependent retardation of tumor growth without toxicity. In another study, quercetin dose-dependently suppressed hepatocyte growth factor (HGF)-mediated melanoma cell migration and invasion by the inhibition of c-Met phosphorylation, reduced c-Met homo-dimerization and decreased c-Met protein expression. Moreover, quercetin suppressed the phosphorylation of c-Met downstream molecules including Gab1 (GRB2-associated-binding protein 1), FAK (Focal Adhesion Kinase) and PAK (p21-activated kinases) [81]. Quercetin was also analyzed in order to observe its effects on A375SM and A375P human melanoma cells. In a dose-dependent manner, quercetin decreased the viability and proliferation of A375SM cells and induced apoptosis without an effect on A375P cells. In vivo, a decrease in the tumor’s volume was reported, indicating that quercetin retards the growth of A275SM melanoma cells through apoptosis, making this polyphenol an effective agent against melanoma [82]. Quercetin also reduces proliferation and induced apoptosis of B16 melanoma cells in vitro [83]. Conversely, quercetin derivatives such as quercetin-3-glucuronide were evidenced to possess antimelanogenesis properties in human keratinocytes and melanoma cells through NF-κB and AP-1 pathways [84]. The internal structure of polyphenols is also significant. It has been evidenced that the methylation of hydroxyl groups increases their cytotoxic properties [73], but Krenn and Paper [85] demonstrated that nonmethylated isoflavones such as genistein and daidzein demonstrate higher antiangiogenic activity than the methylated compounds such as biochanin A and formononetin. The glycosylated derivatives of flavonoids exert worse cytotoxic properties than aglycones, while the total number of -OH groups is also thought to correlate unfavorably with the molecule’s cytotoxic potential. However, the position of the hydroxyl group is of significance in this aspect. The potential anticancer properties are enhanced by the appearance of free hydroxyls in the A-ring, especially at the C5 position [73], or the methylation of -OH groups in the B-ring. When the number of -OH groups in the C- and B-rings is greater, then the cytotoxic effects of the compounds seem to be worse, but the hydroxylation at the 3′ position of the B-ring does not appear to have any effect on the intensity of anticancer action. 

Taking into account the above, the application of white clover flowers could be an innovative approach in the production of functional muffins with potential low glycemic response and enhanced antioxidant and antiproliferative properties characterized by an increased number of polyphenols, especially flavonoids, which are proven to act in an antiproliferative manner on multiple types of cancers, including melanoma skin tumors, while maintaining the organoleptic properties acceptable and desirable to potential consumers. 

## 4. Materials and Methods

### 4.1. Muffin Preparation

Fresh samples of the mature flower buds (*Trifolium repens* L.) were gathered from non-polluted countryside areas of southern Poland, frozen and freeze dried with lyophilizer (ALPHA 1-4, Martin Christ, Osterode am Harz, Germany). Then, the lyophilisate was minced in a laboratory mincer to obtain a sample not bigger than 0.5 mm (Knifetec Sample Mill 1095, FOSS Tescator, Hillerød, Dennamrk ) and used in that form as a bakery ingredient.

The dough components were as follows (Table 6): wheat flour, type 450 (250 g) (“Złote Pola”, Polish Plants Grains S.A., Krakow, Poland), rapeseed oil (100 g) (“Wyborny”, F.H.P.U. “Marlibo” Z.P.Chr. Czekaj Jacek, Bolesław, Poland), milk with 3.2% fat (230 g) (“Mleczna Dolina, District Dairy Cooperative, Łowicz, Poland), cacao (20 g) (“LOTTE Wedel” Sp. z.o.o., Warszawa, Poland), saccharose (150 g) (“Królewski” Südzuker Polska S.A., Wroclaw, Poland), eggs (65 g) (“Ale jaja” Poultry firm Woźniak Sp. z o.o., Rawicz, Poland), baking powder (8 g) (Dr. Oetker Poland Sp. z o.o., Gdansk, Poland) and baking soda (2 g) (Dr. Oetker Poland Sp. z o.o., Gdansk, Poland). The freeze-dried flower pods were added to the muffin dough instead of wheat flour in the following amounts: (1) 2.5% (6.25 g); (2) 5% (12.5 g); (3) 7.5% (18.75 g); (4) 10% (25 g). After mixing, the ingredients were transferred into a silicone form, baked (180 °C, 20 min) and cooled down. In such a form, the organoleptic properties of the muffins were analyzed. The rest of the muffins were freeze dried with lyophilizer (ALPHA 1-4, Martin Christ, Germany) and then minced in a laboratory mincer to obtain a sample not bigger than 0.5 mm (Knifetec Sample Mill 1095, FOSS Tescator, Sweden).

### 4.2. Methods

#### 4.2.1. Organoleptic Assessment

Following Polish and EU standards [86,87,88,89], a panel of 20 trained people assessed the baked muffins. Each participant was provided with a set of labeled samples and evaluation forms using a 9-point hedonic scale: 1—extremely dislike, 2—strongly dislike, 3—moderately dislike, 4—dislike a bit, 5—neither like nor dislike, 6—like a bit, 7—moderately like, 8—strongly like, 9—extremely like. Product characteristics such as color, shape, aroma, crumb elasticity and taste were analyzed. Water was available for the panelists in order to flush the mouth between analyses. The study protocol was approved by the local Bioethics Committee (137/2023).

#### 4.2.2. Chemical Composition

The dry matter (DM), protein, fat and ash contents of the muffins were determined using standard methods according to the recommendations of the Association of Official Analytical Chemists (AOAC) [90]. Moisture was analyzed by the oven-drying method (AOAC method 940.26) using a laboratory drier (SML 30/250, Zalmed, Warszawa, Poland); ash was analyzed by combustion (550 °C) of known weights of samples in a muffle furnace (FCF5SH, Czylok, Jastrzębie-Zdrój, Poland) (AOAC method 930.05); crude fat was analyzed by the Soxtec automated extraction method (AOAC method 930. 09) [78] using the Soxtec Avanti 2050 Auto Extraction Foss Unit (Tecator, Hillerød, Sweden); protein (N × 6.25) was determined by the Kjeldahl method using a FOSS Digester and Autodistillation Unit, KjeltecTM 8200 (Tecator Foss, Hillerød, Sweden) (AOAC method 978.04); total dietary fiber was analyzed using a commercial kit (K-TDFR, Megazyme International Ireland, Bray Business Park, Bray, Co., Wicklow, Ireland). Carbohydrate content was calculated by difference: 100 − (moisture + ash + protein + fat).

#### 4.2.3. Preparation of the Methanol–Acetone Extracts

Fresh samples (5 g) were subjected to extraction with a mixture of 80% acidified methanol (methanol HCl 2% (95/5 *v*/*v*) (Sigma-Aldrich, St. Louis, MO, USA) for 2 h at 25 °C and then centrifugation (1500× *g*, 15 min). The supernatant was retained and the precipitate was re-extracted with 40 mL of 70% acetone solution (POCH, Gliwice, Poland) for the next 2 h and then centrifuged (1500× *g*, 15 min). The supernatants from both sets of centrifugations were merged and further analyzed.

#### 4.2.4. Total Polyphenols

Total polyphenols in the tested muffins were measured according to the procedure described by Swain and Hillis [91] using Folin–Ciocâlteu reagent (Sigma-Aldrich, St. Louis, MO, USA). Prepared methanol–acetone extracts (Section 4.2.3.) were treated with Folin–Ciocâlteu reagent at room temperature (25 °C) and the absorbance was recorded at 760 nm in a spectrophotometer (Spectro 2000RS, Labomed, Inc., Los Angeles, CA, USA). The content of polyphenols is given in mg gallic acid per 100 g dm.

#### 4.2.5. Determination of ABTS Activity

The antioxidant potential of the studied muffins was measured according to the method proposed by Re et al. [92] using ABTS (2,2-azinobis) (3-ethyl-beznothaizoline-6-sulphonic acid) (Sigma-Aldrich, St. Louis, MO, USA). The 0.5 mL portion of the methanol–acetone extract (Section 4.2.3.) was made up to 1 mL with methanol, then 2 mL of free radical ABTS reagent was poured in. The solution was left to incubate at 30 °C for 6 min. The absorbance of the sample was recorded in a spectrophotometer (Spectro 2000RS, Labomed, Inc.) at 734 nm.

#### 4.2.6. Determination of FRAP 

The FRAP (Ferric-Reducing Ability of Plasma) assay was conducted by the adapted procedure of Benzie and Strain [93] as amended by Bartoń, Fołta and Zachwieja [94]. The work solution for FRAP testing was made as a mixture of (1) acetic acid buffer, pH = 3.6; (2) TPTZ (2,4,6-tris(2-pyridyl)-s-triazine (Sigma-Aldrich, St. Louis, MO, USA); and (3) ferric trichloride hexahydrate (FeCl_3_-6H_2_O) (Sigma-Aldrich, St. Louis, MO, USA) in the following ratio: 2:1:1. Reduction of ferric (FeIII) ions to ferrous (FeII) ions resulted in an intense blue color of the FeII-TPTZ complex with an absorbance maximum at 593 nm. A multidetection microplate reader (Synergy 2, Bio-Tek, Winooski, VT, USA) was used for the assay. The test was performed at 37 °C. A 0.400 mL amount of acetate buffer was pipetted into each well, while 0.050 mL of methanol–acetone extract (Section 4.2.3.) was dispensed, followed by the addition of 0.2 mL working FRAP reagent. Absorbance was read at approx. 1.5 min intervals within 60 min of the addition of the working FRAP reagent. The procedure was calibrated using ferrous sulphate heptahydrate (Fe_2_SO_4_-7H_2_O) standard solution (Sigma-Aldrich, St. Louis, MO, USA) in the concentration range of 0–1.5 mmol·L^−1^.

#### 4.2.7. Reversed-Phase High-Performance Liquid Chromatography (RP-HPLC) Analysis of Flavonoids and Phenolic Acids

Twenty-five grams (25 g) of dry mass was extracted with 50 mL of methanol in an ultrasonic bath (POLSONIC Palczyński Sp. J., Warsaw, Poland) for 1 h at 30 °C. After evaporation, dry extracts were dissolved in 10 mL of methanol (HPLC gradient grade) and filtered through Millipore membrane filters with a pore size of 0.22 µm. RP-HPLC analyses were performed according to Sulkowska-Ziaja et al. [95] on a Merck-Hitachi liquid chromatograph (LaChrom Elite) with diode array detector (DAD) L-2455 and Purospher^®^ RP-18e (250 × 4 mm/5 mm) column. Analyses were run at 25 °C using a mobile phase consisting of A—methanol, B—methanol and 0.5% acetic acid at a ratio of 1:4 (*v*/*v*). The gradient was as follows: 100% B for 0–20 min; 100–80% B for 20–35 min; 80–60% B for 35–55 min; 60–0% B for 55–70 min; 0% B for 70–75 min; 0–100% B for 75–80 min; 100% B for 80–90 min at a flow rate of 1 mL min^−1^. Detection was performed in the λ range of 200–400 nm, quantitative calculations were performed at λ = 254 nm. Identification was performed by comparison of peak retention times with authentic reference compounds and co-chromatography with standards. Identification was achieved by comparison of the retention times of the peaks with authentic reference compounds and co-chromatography with standards. Quantification was performed by measuring the peak area with reference to the standard curve derived from five concentrations (0.03125 to 0.5 mg mL^−1^). The standards were as follows: caffeic acid, chlorogenic acid, cinnamic acid, ellagic acid, gallic acid, gentizic acid, isoferulic acid, neochlorogenic acid, *o*-coumaric acid, protocatechuic acid, rosmarinic acid, salicylic acid, sinapic acid, syringic acid, apigenin, apigetrin (apigenin 7-glucoside), hyperoside (quercetin 3-O-galactoside), formononetin, isoquercetin (quercetin 3-O-glucoside), isorhamnetin, kaempferol, luteolin, myricetin, populnin (kaempferol 7-O-glucoside), robinin (kaempferol 3-O-robinoside-7-O-rhamnoside), quercetin, quercitrin (quercetin 3-O-rhamnoside), rhamnetin, rutoside and vitexin from Sigma Aldrich (St Louis, MO, USA); *p*-coumaric acid, vanillic acid, ferulic acid and *p*-hydroxybenzoic acid from Fluka (Bucha, Switzerland); caftaric acid, cryptochlorogenic acid, isochlorogenic acid, catechin, epigallocatechin, epicatechin gallate, epicatechin, epigallocatechin gallate and cinaroside (luteolin 7-O-glucoside) from ChromaDex (Irvine, CA, USA); 4-O-feruloylquinic acid, apigetrin (apigenin 7-O-glucoside), apigenin 7-O-glucuronide, astragalin (kaempferol 3-O-glucoside), avicularin (quercetin 3-*O*-α-L-arabinofuranoside) and trifolin (kaempferol 3-O-galactoside) from ChemFaces (Wuhan, China).

#### 4.2.8. Starch Nutritional Fractions and the Value of the In Vitro Glycemic Index

Total starch (TS) was measured using a commercial kit (K-TSTA 07/11, Megazyme International Ireland, Bray Business Park, Bray, Co., Wicklow, Ireland). Briefly, a freshly ground sample (100 mg) was treated with a mixture of Thermostable α-amylase (3000 U/mL) and amyloglucosidase (3300 U/mL) for 30 min at 50 °C. The hydrolyzed glucose was determined using the GOPOD reagent with glucose oxidase and peroxidase. The absorbance of the resulting colored products was measured at 510 nm in a spectrophotometer (Spectro 2000RS, Labomed Company, Inc.) and the amount of TS was calculated. The concentrations of slowly digestible starch (SDS), rapidly digestible starch (RDS) and free glucose (FG) were determined by the method described by Englyst et al. [96] and modified by Chung et al. [97]. The resulting enzyme mixture was freshly made, and the following components were added: porcine pancreatic α-amylase (P-7545, 8 × USP specifications, Sigma, St. Louis, MO), amyloglucosidase (3300 U·mL^−1^, Megazyme International Ireland Ltd., Bray, Ireland) and invertase (I-4504, ≥300 units-mg^−1^ solid, Sigma, St. Louis, MO, USA). The muffins were weighed in a quantity containing 100 mg TS and 4 mL 0.5 M sodium acetate buffer (pH 5.2). The enzyme mixture and glass beads were added to the tube and incubated in a shaking water bath (37 °C, 200 shakes/min). The released glucose was measured after 20 min and 120 min using GOPOD reagent at 510 nm (K-GLOX, International Ireland, Bray Business Park, Bray, Co., Wicklow, Ireland) in a spectrophotometer (Spectro 2000RS, Labomed company, Inc.). The final result was the starch digestion index (SDI). The amounts of RDS, RAG and SDS and the SDI value (reffered as to GI in vitro) of the samples were calculated using the following equations:

RAG (g·100 g^−1^ dm) = G_20_ + FG (1)

RDS (g·100 g^−1^ dm) = (G_20_ − FG) × 0.9 (2)

SDS (g·100 g^−1^ dm) = (G_120_ − G_20_) × 0.9 (3)

(4) $$SDI\;(\%)=\frac{RDS}{TS}\;\times \;100$$

#### 4.2.9. Cell Cultures

##### Cell Lines

The following cell lines were used for in vitro testing of potential anticancer properties:

- A line BJ (control)—a line of human normal fibroblasts, obtained from the American Type Culture Collection (ATCC), cultured using Eagle’s Minimum Essential Medium (EMEM) (RPMI 1640, Sigma-Aldrich, St. Louis, MO, USA) with penicillin antibiotic and 10% fetal bovine serum (FBS) (F9665, Sigma-Aldrich, St. Louis, MO, USA);
- A line A375—a human skin cancer (melanoma) line, obtained from the American Type Culture Collection (ATCC), cultured using Dulbecco’s Modified Eagle’s Minimum Essential Medium (DMEM) (SLM 202, Sigma-Aldrich, St. Louis, MO, USA) with penicillin antibiotic and 10% fetal bovine serum (FBS) (F9665, Sigma-Aldrich, St. Louis, MO, USA).

##### Cells Preparation

Cells were cultured in an incubator with controlled conditions (temp. 37 °C, 95% humidity, 5% CO_2_) in cell culture medium according to the manufacturer’s recommendations. Twice a week, or as needed to ensure cell confluence did not exceed 80%, a passage of cells was performed. The medium was poured off, the cells were washed with 1 mL of PBS solution (Sigma-Aldrich, St. Louis, MO, USA) and 0.5 mL of trypsin (Sigma-Aldrich, St. Louis, MO, USA) was added. They were incubated for about 5 min until the cells detached from the medium, then the trypsin was inactivated by adding 1 mL of medium. A subcultivation ratio of 1:4 was used, where the cells were transferred to another bottle (T-25 Sarstedt, Nümbrecht, Germany) and 5 mL of fresh medium was added. Cultures were continued at 37 °C and 5% CO2 until the desired number of cells was reached. 

##### Cells Seeding

After counting the cells using an automatic counter from Bio-Rad (model TC20 TM), they were seeded into 96-well plates (Corning, NY, USA). A solution of 14.4 mL cell suspension mixed with fresh medium was prepared. Each cell line was seeded into individual wells at a density of 20,000 cells per well in 150 μL of medium. The plates were then incubated for 24 h at 37 °C in an atmosphere of 5% CO_2_.

##### Adding Experiments Conditions

After 24 h, the medium was replaced with fresh medium containing 0.5 mg/mL, 1.5 mg/mL and 2.5 mg/mL muffin extracts. Cells cultured in medium without an extract were used as a control. Each condition was applied in triplicate. The cells were then incubated with the extracts for an additional 48 h.

##### Cytotoxicity Assay

The effect of cytotoxicity of extracts on cells was assessed using the Cytotoxicity Detection Kit (LDH) (Roche, Basel, Switzerland) according to the manufacturer’s protocol. Cytotoxicity was determined after 48 h. Assessment of LDH activity in the cell supernatant was the measure of the toxicity of the investigated substance in relation to cultured cells. It was a colorimetric method and the assays were performed using an ELISA microplate reader, the Multiskan GO (Thermo Scientific, Waltham, MA, USA). 

##### Cell Proliferation Assay

Cell proliferation was evaluated at 48 h of incubation using the Cell Proliferation ELISA BrdU kit (Roche, Basel, Switzerland) according to the manufacturer’s protocol. A 10 μL amount of BrdU labeling solution was added to each well and incubated at 37 °C for 2 h. After incubation, the solutions were removed, and 100 μL of fixative (FixDenat) was added to each well and incubated for 30 min at room temperature. The fixative was then removed, and 50 μL of anti-BrdU-POD working solution was added and incubated for 90 min at room temperature. Following this, the wells were washed three times with 25 μL of washing solution. Subsequently, 50 μL of substrate solution was added to each well and incubated for 20 min at room temperature. The absorbance was measured at 370 nm using a Thermo Scientific Multiskan GO spectrophotometer (Thermo Scientific, Waltham, MA, USA).

### 4.3. Data Analyses

The results are presented as mean values with standard deviations of triplicates. One-way analysis of variance was applied in order to assess the influence of different white clover flower additions to the tested muffins on the assessed parameters. The Duncan and RIR Tukey tests were used in order to evaluate the significance of differences at a level of *p* < 0.05. All the calculations were carried out using Statistica v.10 software (Statsoft, Inc., Tulsa, OK, USA).

## 5. Conclusions

The overall ratings of the tested muffins with 2.5%–7.5% addition of white clover flowers signified great acceptance, while 10% enrichment was assessed by the panelists as worse. All the *Trifolium-repens*-enriched muffins had significantly higher free-radical-scavenging potential (*p* < 0.05) in comparison to the control samples and contained phenolic acids and flavonoids, naturally presented compounds with proven antidiabetic, antioxidant and anticancer properties. The reduced viability of A375 melanoma cells line in the in vitro tests supported the antiproliferative properties of supplemented muffins. The glycemic response as measured by the content of nutritionally important starch fractions and the starch digestibility index was favorably and statistically significantly reduced upon an addition of lypholized flowers of *Trifolium repens* (*p* < 0.05), resulting in a snack product with a possible low glycemic index. This is especially advantageous for the people suffering from type 2 diabetes and those who would like to protect themselves against the development of this disease. In this regard, the muffins enriched with the white clover flowers could constitute a promising novel snack food, as proven in this study of their functional properties. The continuation of the research is relevant regarding the influence of the designed muffins on the glycemic index and the bioavailability of the polyphenolic compounds tested on human volunteers and the determination of some natural, non-nutritional plant substances, including saponins, cyanogenic glycosides and tannins concurrently with a more thorough inspection of the possible mechanisms by which the muffins reduce the proliferation of cancer cells, including the extension of the research scope to other tumor cell lines

## Figures and Tables

**Table 1:** Organoleptic characteristics of muffins enriched with white clover flowers (*Trifolium repens* L.).

	Quality Parameters						
	Color	Shape	Flavor	Elasticity	Tastiness	Total	Overall Assessment
Muffins with Flowers	Mean ± sd						
Control	8.40 ^a^ ± 0.7	7.45 ^a^ ± 1.4	8.10 ^a^ ± 0.6	7.80 ^a^ ± 0.8	8.00 ^a^ ± 1.08	7.95 ^a^ ± 1.0	Strongly like
2.5%	8.15 ^a^ ± 0.8	7.35 ^a^ ± 1.0	8.00 ^a^ ± 0.7	7.65 ^a^ ± 0.9	8.05 ^a^ ± 0.8	7.84 ^a^ ± 0.9	Strongly like
5%	8.30 ^a^ ± 0.7	7.40 ^a^ ± 1.4	7.90 ^a^ ± 0.8	7.85 ^a^ ± 1.1	7.45 ^b^ ± 0.7	7.78 ^ab^ ± 1.0	Strongly like
7.5%	7.95 ^ab^ ± 1.2	7.35 ^a^ ± 1.4	7.55 ^a^ ± 0.7	7.80 ^a^ ± 1.0	6.90 ^c^ ± 0.9	7.51 ^b^ ± 1.1	Strongly like
10%	7.55 ^b^ ± 0.9	7.20 ^a^ ± 0.7	6.05 ^b^ ± 1.6	7.45 ^b^ ± 0.8	5.85 ^d^ ± 0.9	6.82 ^c^ ± 1.2	Moderately like

The results are presented as mean ± sd. The values with varied letters in columns are significantly different at *p* < 0.05.

**Table 2:** Chemical composition of muffins enriched with white clover flowers (*Trifolium repens* L.).

Analyzed Parameters/Samples	Dry Matter (%)	Carbohydrates (g·100 g^−1^ dm)	Available Carbohydrates (g·100 g^−1^ dm)	Protein (g·100 g^−1^ dm)	Fat (g·100 g^−1^ dm)	Dietary Fiber (g·100 g^−1^ dm)	Ash (g·100 g^−1^ dm)
*Trifolium* *repens* L. powder	92.90 ^f^ ± 0.1	-	-	20.48 ^f^ ± 0.2	2.21 ^c^ ± 0.1	5.19 ^f^ ± 0.0	11.83 ^e^ ± 0.0
control	80.84 ^e^ ± 0.2	42.56 ^b^ ± 0.2	40.94 ^b^ ± 0.0	11.60 ^b^ ± 0.1	23.95 ^a^ ± 0.1	1.62 ^a^ ± 0.0	2.73 ^a^ ± 0.0
2.5%	79.85 ^d^ ± 0.0	42.99 ^b^ ± 0.0	40.92 ^b^ ± 0.0	10.88 ^a^ ± 0.1	23.22 ^a^ ± 0.1	2.07 ^b^ ± 0.0	2.76 ^a^ ± 0.0
5%	75.52 ^c^ ± 0.4	33.62 ^c^ ± 0.4	31.29 ^c^ ± 0.0	12.19 ^c^ ± 0.1	26.66 ^b^ ± 0.3	2.33 ^c^ ± 0.0	3.05 ^b^ ± 0.0
7.5%	72.54 ^b^ ± 0.3	28.74 ^c^ ± 0.3	26.12 ^c^ ± 0.0	12.93 ^d^ ± 0.1	27.40 ^b^ ± 0.2	2.62 ^d^ ± 0.0	3.47 ^c^ ± 0.0
10%	68.21 ^a^ ± 0.1	15.04 ^d^ ± 0.1	11.95 ^d^ ± 0.0	15.29 ^e^ ± 0.1	33.16 ^d^ ± 0.9	3.09 ^e^ ± 0.0	4.72 ^d^ ± 0.0

The results are presented as mean ± sd. The values with varied letters in columns are significantly different at *p* < 0.05.

**Table 3:** Starch digestibility of muffins supplemented with white clover flowers (*Trifolium repens* L.).

Analyzed Parameters/Samples	TS (g·100 g^−1^ dm)	FG (g·100 g^−1^ dm)	SDS (g·100 g^−1^ dm)	RDS (g·100 g^−1^ dm)	RS (g·100 g^−1^ dm)	RAG (g·100 g^−1^ dm)
control	42.29 ^a^ ± 0.2	5.09 ^c^ ± 0.1	3.71 ^a^ ± 0.1	22.03 ^d^ ± 0.4	16.54 ^b^ ± 0.5	34.67 ^d^ ± 0.1
2.5%	42.04 ^a^ ± 0.2	4.43 ^b^ ± 0.2	3.58 ^a^ ± 0.4	9.88 ^b^ ± 0.2	28.57 ^d^ ± 0.2	19.86 ^b^ ± 0.2
5%	43.85 ^b^ ± 0.1	4.58 ^a^ ± 0.0	4.75 ^b^ ± 0.3	14.63 ^a^ ± 0.7	24.45 ^a^ ± 0.4	25.43 ^a^ ± 0.8
7.5%	43.27 ^b^ ± 0.1	4.68 ^a^ ± 0.1	4.84 ^b^ ± 0.1	14.33 ^a^ ± 0.5	24.1 ^a^ ± 0.5	25.28 ^a^ ± 0.7
10%	43.62 ^b^ ± 0.1	4.69 ^a^ ± 0.1	6.73 ^c^ ± 0.7	15.80 ^c^ ± 0.6	21.08 ^c^ ± 0.3	26.94 ^c^ ± 0.5

The results are presented as mean ± sd. The values with varied letters in columns are significantly different at *p* < 0.05. TS—total starch; SDS—slowly digestible starch; RDS—rapidly digestible starch; RS—resistant starch; RAG—rapidly available glucose; FG—free glucose.

**Table 4:** Total polyphenols and polyphenolic compound profile of the tested muffins.

	Flowers of *Trifolium repens* L.	Control Muffins	Muffins with White Clover Flowers			
			2.5%	5%	7.5%	10%
Total polyphenols (mg·100 g^−1^ dm)	2409.36 ^e^ ± 35.72	21.26 ^a^ ± 3.78	232.65 ^a^ ± 3.87	292.94 ^b^ ± 2.51	374.32 ^c^ ± 4.10	431.37 ^d^ ± 5.29
Phenyl propanoic acids (µg·100 g^−1^ dm)						
protocatechuic acid	2.286 ^a^ ± 0.20	0.950 ^b^ ± 0.05	0.592 ^c^ ± 0.03	0.762 ^bcd^ ± 0.01	0.991 ^bd^ ± 0.04	1.996 ^e^ ± 0.03
*p*-OH-benzoic acid	7.421 ^a^ ± 0.14	0.195 ^b^ ± 0.00	0.234 ^c^ ± 0.01	0.329 ^cd^ ± 0.00	0.414 ^d^ ± 0.02	0.890 ^e^ ± 0.02
*p*-coumaric acid	29.388 ^a^ ± 0.82	nd	0.564 ^b^ ± 0.07	1.150 ^b^ ± 0.06	1.557 ^b^ ± 0.10	2.925 ^c^ ± 0.11
ferulic acid	9.958 ^a^ ± 0.20	0.037 ^b^ ± 0.00	0.270 ^c^ ± 0.02	0.349 ^bc^ ± 0.01	0.434 ^c^ ± 0.01	0.946 ^d^ ± 0.03
Flavonoids (µg·100 g^−1^ dm)						
quercetin 3-glucoside	371.265 ^a^ ± 17.85	nd	1.777 ^b^ ± 0.12	4.616 ^b^ ± 0.21	8.172 ^b^ ± 0.28	21.189 ^b^ ± 0.24
quercetin-3-α-l-arabino furanoside (avicularin)	36.082 ^a^ ± 5.50	nd	1.248 ^b^ ± 0.02	1.408 ^b^ ± 0.00	1.255 ^b^ ± 0.01	2.643 ^b^ ± 0.04
kaempferol-3-O-glucoside (astragalin)	61.480 ^a^ ± 1.33	nd	1.140 ^b^ ± 0.05	1.632 ^b^ ± 0.05	2.079 ^bc^ ± 0.03	3.669 ^c^ ± 0.06
kaempferol 3-rhamnoside	25.962 ^a^ ± 0.73	nd	1.069 ^b^ ± 0.01	1.164 ^b^ ± 0.00	1.416 ^b^ ± 0.01	2.847 ^c^ ± 0.12

The results are presented as mean ± sd. The values with varied letters in superscript in rows are significantly different at *p* < 0.05; nd—not detected.

**Table 5:** Cytotoxicity and proliferation assay after 48 h incubation of muffins on BJ human normal fibroblasts and A375 human melanoma cancer cells.

			Mean Value [%] ± Standard Deviation		
Extract Type	Extract [mg/mL]		Cytotoxicity ^1^	Proliferation ^1^ (as % of Control Sample)	
		BJ Cell Line	A375	BJ Cell Line	A375
*Trifolium repens* L.	0.5	13.51 ^ab^ ± 4.99	13.10 ^a^ ± 0.93	94.13 ^a^ ± 2.52	68.36 ^b^ ± 4.48
	1.5	14.84 ^ab^ ± 4.87	12.16 ^a^ ± 1.13	96.90 ^a^ ± 2.56	36.96 ^a^ ± 0.56
	2.5	13.77 ^ab^ ± 8.69	12.76 ^a^ ± 0.37	94.13 ^a^ ± 3.02	38.90 ^a^ ± 0.49
	*x̅*	14.04	12.67	95.05	48.07
Muffins with 7.5%	0.5	13.06 ^ab^ ± 5.00	13.74 ^a^ ± 3.03	170.69 ^bd^ ± 3.53	54.55 ^ab^ ± 4.42
	1.5	15.84 ^a^ ± 1.36	13.97 ^a^ ± 1.05	122.57 ^ac^ ± 2.08	43.28 ^ab^ ± 3.87
	2.5	15.30 ^a^ ± 1.10	14.25 ^a^ ± 1.50	156.69 ^bd^ ± 1.54	41.22 ^ab^ ± 4.78
	*x̅*	14.73	13.99	149.89	46.35
Muffins with 2.5%	0.5	13.01 ^ab^ ± 1.31	13.75 ^a^ ± 1.36	155.85 ^bd^ ± 3.41	114.45 ^cd^ ± 6.48
	1.5	13.22 ^ab^ ± 2.24	14.70 ^a^ ± 2.42	211.00 ^e^ ± 4.47	94.65 ^c^ ± 8.23
	2.5	14.16 ^ab^ ± 4.47	13.83 ^a^ ± 1.39	137.86 ^bc^ ± 2.44	62.80 ^ab^ ± 4.46
	*x̅*	13.46	14.1	168.24	90.63
Control muffins	0.5	7.95 ^b^ ± 6.41	13.35 ^a^ ± 2.70	122.33 ^ac^ ± 1.47	126.62 ^d^± 3.63
	1.5	12.39 ^ab^ ± 4.84	14.33 ^a^ ± 1.21	180.07 ^d^ ± 1.98	118.83 ^cd^ ± 2.64
	2.5	13.45 ^ab^ ± 4.64	14.11 ^a^ ± 1.97	144.37 ^bc^± 1.41	120.93 ^cd^ ± 4.38
	*x̅*	11.26	13.93	148.92	122.12

^1^ The results are presented as mean ± sd. The values with varied letters in columns are significantly different at *p* < 0.05.

**Table 6:** The recipe formulation of different kinds of muffin enriched with white clover flowers (*Trifolium repens* L.).

	Dough Ingredients [g]								
Muffin Kinds	Wheat Flour	Rapeseed Oil	Milk	Saccharose	Eggs	Baking Powder	Baking Soda	Cacao	Lypholized Flower Buds
Control	250	100	230	180	6	8	2	20	0
2.5%	243.75	100	230	180	6	8	2	20	6.25
5%	237.50	100	230	180	6	8	2	20	12.5
7.5%	231.25	100	230	180	6	8	2	20	18.75
10%	225.00	100	230	180	6	8	2	20	25.00

## Data Availability

Data are unavailable due to privacy or ethical restrictions.
